# Impact of Heat Stress on Blood, Production, and Physiological Indicators in Heat-Tolerant and Heat-Sensitive Dairy Cows

**DOI:** 10.3390/ani13162562

**Published:** 2023-08-09

**Authors:** Xiaoyang Chen, Hang Shu, Fuyu Sun, Junhu Yao, Xianhong Gu

**Affiliations:** 1 State Key Laboratory of Animal Nutrition, Institute of Animal Science, Chinese Academy of Agricultural Sciences, Beijing 100193, China; bmwa123690@163.com (X.C.); caassfy@163.com (F.S.); 2 College of Animal Science and Technology, Northwest A&F University, Xianyang 712100, China; yaojunhu2004@sohu.com; 3 Agricultural Information Institute, Chinese Academy of Agricultural Sciences, Beijing 100086, China; hang.shu@doct.uliege.be

**Keywords:** heat stress, dairy cows, blood, physiological indicators, production, heat-tolerant, heat-sensitive

## Abstract

**Simple Summary:**

Heat stress in dairy cows can be assessed based on induced responses from behavioral, physiological, and health aspects. We can use reaction norm models to quantify the individual responses of cows across the trajectory of THI during thermal environments to identify heat-tolerant and heat-sensitive cows. In this study, we investigated the effects of heat stress on blood, production, and physiological indicators of heat-tolerant and heat-sensitive cows. In conclusion, heat stress increased the cortisol in heat-sensitive cows. Additionally, heat-tolerant cows may improve their adaptability to thermal environments of cows by upregulating their respiration rate rapidly.

**Abstract:**

Heat stress affects production and health in cows severely. Since it is difficult to define heat-tolerant animals, studies of response to heat stress are important for understanding dairy cows’ health and production. However, information on the impact of heat stress on various indicators in heat-tolerant and heat-sensitive cows is sparse. This study aimed to investigate the effects of heat stress (HS) on blood, production, and physiological indicators in heat-tolerant and heat-sensitive cows. A total of 43 dairy cows were used from 9 May to 7 August 2021, under Temperature–Humidity Index (THI) measurements that ranged from 65.9 to 86.7. We identified cows that were tolerant or sensitive to HS based on the slope of the response of physiological and production traits against THI during the HS period by using a clustering method. After HS, serum glucose (Glu), cortisol (COR), 5-hydroxytryptamine (5-HT), and interleukin-6 (IL-6) levels of cows in the heat-tolerant group were lower than in the heat-sensitive group (*p* < 0.05). With THI as the predictor, the R^2^ for predicting respiration rate (RR) and body surface temperature (BT) in heat-tolerant cows was 0.15 and 0.16, respectively, whereas the R^2^ for predicting RR and BT in heat-sensitive cows was 0.19 and 0.18, respectively. There were low to moderate, positive correlations between RR, BT, and MY with THI, with Pearson correlation coefficients ranging from r = 0.11 to 0.4 in the heat-tolerant group, and from r = 0.24 to 0.43 in the heat-sensitive group. There was a low positive correlation between VT and THI, with a Spearman correlation coefficient of r = 0.07 in the heat-sensitive group. The heat-tolerant dairy cows had lower MY losses and had lower MY (*p* = 0.0007) in mixed models. Heat-tolerant cows with low-stress levels, through upregulating RR rapidly, increased their adaptability to thermal environments. They have better thermoregulation capability; the hypothalamic–pituitary–adrenal (HPA) axis regulated the thermoregulatory in animals by releasing a variety of neurotransmitters and hormones.

## 1. Introduction

Global climate change is becoming increasingly visible in the world’s agriculture, and the rising heat load has a substantial impact on the production and welfare of dairy cows. Depending on the time of year, heat stress (HS) can occur in any climate zone [1]. The HS-related decreased performance is commonly linked to the summer months; nevertheless, the detrimental impacts might persist into the fall months, even if cows are not experiencing heat stress [2,3]. Heat stress has an impact on a variety of dairy production parameters, including reduced milk production, impaired reproductive performance, increased risk of lameness and mastitis, and even death [4]. In China, THI-based milk yield (MY) losses were up from 0.7 to approximately 4 kg per cow per day in July 2016, these losses are projected to increase from 1.5 to 6.5 kg in 2050 and 2 to 7.2 kg in 2070 [5]. All total, the dairy industry experiences substantial losses due to HS. Dairy cow production demonstrates an antagonistic association between MY and heat-resistance at any level of production. When compared to lower-producing or primiparous cows, multiparous cows and cows with higher MY are more vulnerable to the effects of HS [6,7]. It is extremely difficult and time-consuming to breed high-yielding and heat-tolerant cows. Therefore, exploring the difference between cows that are heat-tolerant and those that are heat-sensitive appears to be important to facilitate the development of robust and cost-effective HS recovery strategies.

Measurement at the animal level is important to manage HS due to considerable animal-to-animal variance and differences in responses when subjected to heat stress [8]. Heat stress can be assessed based on variables that reflect behavioral, physiological, and health aspects of the animals when they fail to cope with deleterious HS effects and also based on their previous health status [9]. Respiration rate (RR) increase is an early sign of the start of heat stress, with a high RR representing a greater capability for cooling [10]. Body temperature increase is another important measure of heat stress, representing a summary of thermoregulatory responses [6]. Body temperature can be measured vaginally and rectally, with an excellent correlation between the two methods [11]. According to Barros et al. [12], infrared thermography was a useful, accurate, and non-invasive approach for measuring body surface temperature (BT) in the orbital region, left flank, right flank, and scrotum in buffaloes, with a positive association with the temperature-humidity index (THI). Carabano et al. [13] reported that the slope of production trait trends and somatic score against ambient temperatures could be further evaluated as selection criteria to determine heat tolerance. Amamou et al. [14] used mixed models to evaluate the effects of THI on physiological and production traits in two groups of cows with varied heat sensitivities, and found that cows that qualified to be heat-tolerant tended to have higher slopes (to a unit increase in THI) in RR, BT, and RT (vaginal temperature) and lower slopes, with almost no decay in milk yield compared to cows that qualified to be heat-sensitive. In summary, by considering these various measures and modeling approaches, using both physiological and production trait indicators, it becomes possible to evaluate a cow’s heat-tolerance and sensitivity, enabling better management and understanding of heat stress effects in cows.

Heat stress leads to changes in endocrine secretions and activates stress responses in cows [15]. HS activates the hypothalamic–pituitary–adrenal (HPA) axis which is the predominant endocrine regulator for stress response in animals. The activation of the HPA axis stimulates the secretion of the classical stress marker, cortisol, and further increases the circulating glucose [15]. The increase in circulating glucose during HS is essential in allowing ruminants to cope with heat stress [16]. Cortisol can increase the HPA axis sensitivity to later stress challenges via negative feedback pro-inflammatory cytokines, such as interleukin-6 (IL-6) [17]. These endocrine secretions can influence the production and activity of HSPs [18]. Thus, endocrine secretions can stimulate the synthesis and expression of HSPs in cells and tissues as a protective mechanism; furthermore, HSPs can also have regulatory effects on the immune system. One heat shock protein, HSP-70, is highly inducible and the most sensitive to HS in ruminants, and its expression escalates in proportion to the severity of HS [19]. During HS, there is an increase in heat shock protein HSP-90, which is observed in cattle, buffalo, broilers, sheep and goats [15]. A crosstalk between the endocrine secretions, HSPs, and the immune system is established during heat stress, with HSPs playing both a stimulatory and regulatory role. However, the effects of the thermal environment on endocrine secretory processes in heat-tolerant and heat-sensitive cows are complex and not fully understood.

Therefore, we hypothesized that it would be possible to screen cows for heat-tolerant or heat-sensitivity by using fewer, but more precise parameters, and that cows with heat-tolerance would experience greater physiological changes compared to cows with heat sensitivity, but with less impact on their endocrine secretory and productive performance. To test this hypothesis, we measured the RR, VT, BT, and MY of dairy cows and THI during thermal environments. We then selected heat-tolerant and heat-sensitive cows by using the slopes of various animal-based indicators against THI. In this context, the objectives of this study were to investigate the changes in blood, production, and physiological indicators in cows with heat-tolerance or heat-sensitivity exposed to HS, and to provide a reliable basis for the development of robust and cost-effective HS recovery strategies.

## 2. Materials and Methods

### 2.1. Animals and Experimental Design

**Figure 1:**
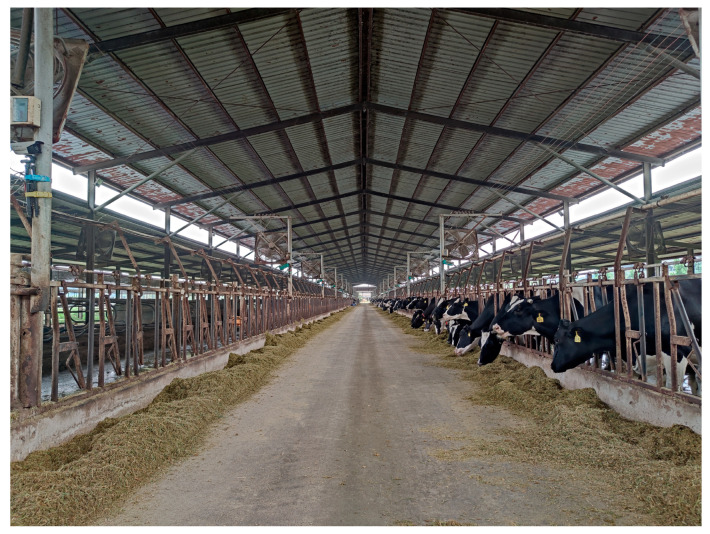
An intensive organic dairy farm in Shandong, China.

The experiment was conducted from May to August, 2021, at an intensive organic dairy farm in Shandong, China (coordinates: 34°50′37″ N, 115°26′11″ E; altitude: 52 m) (Figure 1), which belongs to a temperate continental monsoon climate. Forty-three mid-lactating Holstein cows were selected in the experimental period. Mean daily milk yield, parity, days in milk (DIM), and body condition scoring (BCS) of selected dairy cows were 40.4 ± 5.7 kg/day, 2.6 ± 1.05, 150.0 ± 18.8, and 3.1 ± 0.2, respectively. The experimental barn was mechanically ventilated, contained four pens (15 m × 90 m each, oriented along the north-south longitudinal axis), and had a 400 lying-bed capacity, with concrete floors and no outdoor area. All cows were milked at 08:00, 15:00, and 20:00 (Table 1), and fed (same total mixed ration) at 08:30, 15:30, and 20:30. All cows had free access to water during the experimental period.

**Figure 2:**
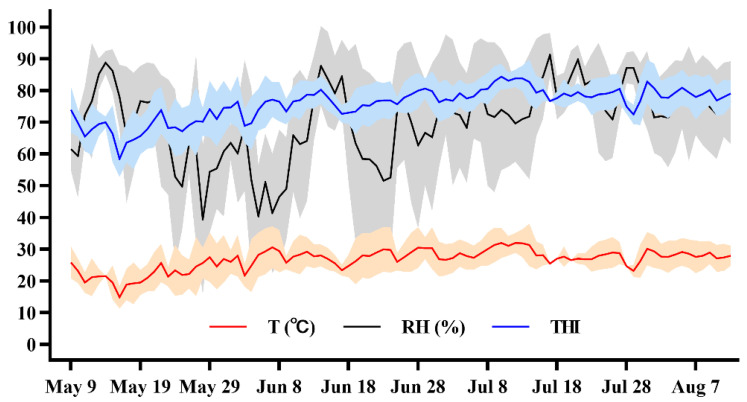
Daily minimum, maximum, and mean environmental ambient temperature (°C), relative humidity (%), and temperature-humidity index (THI) for the duration of environmental heat stress exposure from 9 May to 7 August 2021, at the dairy experiment farm in Shandong.

Environmental parameters were recorded every 10 min with Kestrel 5000 heat stress trackers (Nielsen-Kel-lerman, Boothwyn, PA, USA) to measure ambient temperature (Ta, °C), wet bulb temperature (Tw), dew point temperature (Tdp), relative humidity (RH, %), and wind speed (WS). Daily minimum, maximum, and mean ambient temperature, RH, and THI measured throughout the study are described in Figure 2. THI was calculated using the following equation by the National Research Council [20]: (1.8 × Ta + 32) − [(0.55 − 0.0055 × RH)] × (1.8 × Ta − 26).

### 2.2. Physiological Indicators and Milk Yield Measurement

RR, VT, and BT were used as physiological indicators. A detailed method for measuring the physiological indicators can be found in Shu et al. [21]. Physiological indicators were measured twice a day per cow, and MY was recorded for each cow three times on all test days (Table 2).

### 2.3. Blood Collection and Processing

At the end of the experimental period, 10 mL blood samples were collected from 20 cows (10 cows randomly selected from each group, after clustering, for a total of 20 cows) at 14:00 to 14:30 (neither the feeding nor milking time) into vacutainers (BD vacutainers, Fisher Scientific, Waltham, MA, USA; Table 2). Blood samples were centrifuged at 3000× *g* for 15 min at 4 °C to isolate serum and stored at −80 °C. Blood glucose (GLU) was measured using an AU480 auto-analyzer (Olympus Co., Tokyo, Japan). The BFM-96 multi-tube radioimmunoassay counter (Hefei, China) was used to determine the content of cortisol (COR). The levels of serotonin (5-HT; Bovine 5-HT Elisa Kit; intra- and inter-assay coefficients of variability: CV < 12%, CV < 12%), interleukin 6 (IL-6; Bovine IL-6 Elisa Kit; intra- and inter-assay coefficients of variability: CV < 12%, CV < 12%), heat shock protein 90 (HSP-90; Bovine HSP-90 Elisa Kit; intra- and inter-assay coefficients of variability: CV < 12%, CV < 12%), and heat shock protein 70 (HSP-70; Bovine HSP-70 Elisa Kit; intra- and inter-assay coefficients of variability: CV < 12%, CV < 12%) in the samples were determined by biotin double-antibody sandwich ELISA, following the manufacturer’s instructions. All the above colorimetric data were measured using THERMO Multiskan Ascent (Waltham, MA, USA).

### 2.4. Statistical Analysis

All statistical analyses were performed using SAS (version 9.4, SAS Institute Inc., Cary, NC, USA). All analyses were performed based on the actual measurements of each cow over the entire experimental period. Mixed-effect models were fitted by using PROC MIXED. The models used the data obtained during the HS period for quantifying the individual differences in cows’ tolerance and sensitivity to HS. The models included the fixed effects of the cow location within the day of measurement, parity, days in milk, and age at calving classes; random effects of the individual variability (the intercept, standing for the general component of the parameter, independent of the THI effect and the slope, reflecting the response of the parameter to a unit increase in THI); and the residual error. The dependent variables were RR, BT, VT, and MY.

The clustering of heat-tolerant and heat-sensitive groups was based on the estimated individual slopes of physiological (i.e., RR, VT, and BT) and production indicators to THI from the mixed-effect models. Hierarchical cluster analysis, based on Ward’s method, was performed by using PROC CLUSTER after the standardization of individual slopes. The division into similar groups would allow us to identify dairy cows with a similar response to HS, thus reducing response heterogeneity while increasing intra-group response homogeneity. The clustering result was visualized and interpreted with the factor map from a principal component analysis (PCA) by using PROC PRINCOMP.

Regression analyses were performed by using PROC REG to determine the optimal indicators of selecting heat-tolerant or heat-sensitive dairy cows. All animal-based indica-tors (i.e., RR, VT, BT, and MY) were used as the dependent variables, and the THI as the independent variable. The goodness of fit of the models was evaluated by the coefficient of determination (R^2^) and root mean square error (RMSE). Pearson correlation coefficients were calculated using PROC CORR to explore the linear relationship between THI with physiological and production indicators within the heat-tolerant and heat-sensitive groups. The Student’s t-test was performed with PROC TTEST to identify statistically significant differences between the heat-tolerant and heat-sensitive groups in terms of the physiological, production, and serum indicators. Serum indicators were collected from 10 randomly selected cows in each group, constituting a total of 20 cows (Table 2). Significance and tendency were declared at *p* < 0.05 and 0.05 < *p* < 0.10, respectively.

## 3. Results

### 3.1. Cluster Results and Analysis

**Figure 3:**
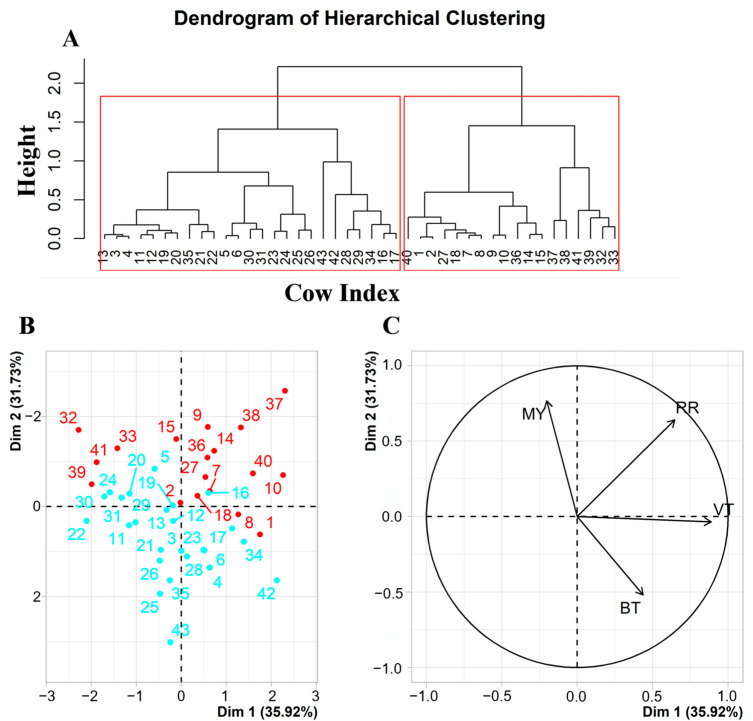
Clustering results visualized by (**A**) a dendrogram plot as well as (**B**) an individual factor map and (**C**) a variables factor map from the principal component analysis. (**B**): the numbers indicate cow codes, and red and blue indicate heat-tolerant and heat-sensitive cows.

The clustering process and results visualized in Figure 3 show that 43 dairy cows were classified into two clusters, with RR, BT, and MY playing a major role in the clustering process. Based on the result of the cluster analysis, 43 dairy cows were classified into two clusters (1 and 2, Table 3). Forty-two percent of the dairy cows belonged to Cluster 1, characterized by increased slopes in production and physiological traits, particularly for MY, RR, VT, and BT shown in Table 3, where the 18 cows from Cluster 1 had increased slopes in production and physiological traits, particularly for MY, RR, VT, and BT (0.26 ± 0.26, 2.03 ± 0.18, 0.02 ± 0.01, and 0.06 ± 0.004). In contrast, the 25 cows from Cluster 2 tended to show a response with flat or negative slopes for MY, RR, VT, and BT of −0.07 ± 0.25, 1.73 ± 0.20, 0.02 ± 0.01, and 0.06 ± 0.004, respectively. Accordingly, Cluster 1 could be qualified as the heat-tolerant group and Cluster 2 could be qualified as the heat-sensitive group.

### 3.2. Linear Regression of Animal-Based Indicators on THI, Correlation between THI and Animal-Based Indicators

Table 4 shows the R^2^ and RMSE for predicting RR, VT, BT, and MY using THI in heat-tolerant and heat-sensitive dairy cows. The R^2^ of RR and BT was higher, relative to the other physiological indicators. The R^2^ for RR and BT was 0.15, 0.16, and 0.19, 0.18 (heat-tolerant and heat-sensitive groups), respectively (Table 4).

**Figure 4:**
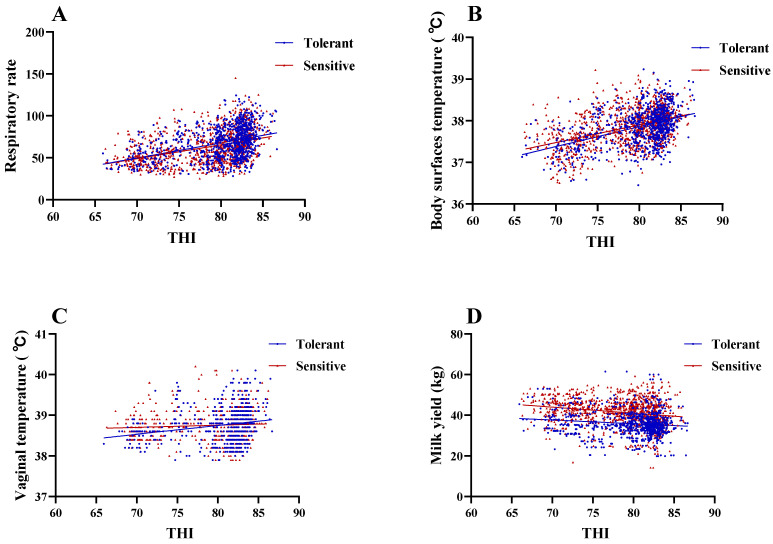
Relationship between respiration rate (**A**), body surface temperature (**B**), vaginal temperature (**C**), and milk yield (**D**) and temperature-humidity index (THI) in dairy cows clustered to heat-tolerant (shown in blue, *n* = 18) or heat-sensitive (shown in red, *n* = 25). Lines represent simple linear regression equations.

**Figure 5:**
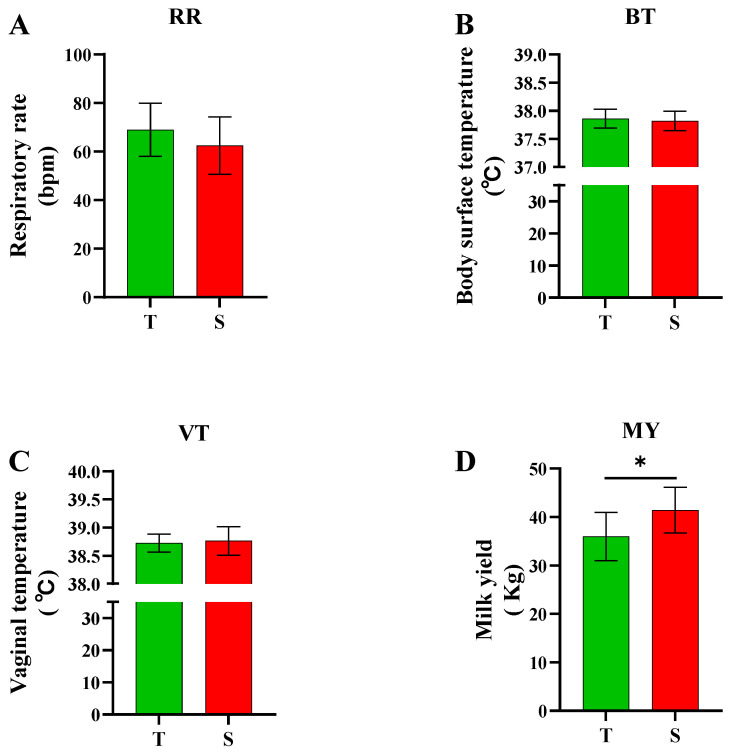
Respiration rate ((**A**); bpm = breaths per minute), body surface temperature (**B**), vaginal temperature (**C**), and milk yield (**D**) of dairy cows from heat-tolerant (T) and heat-sensitive (S) groups. * means statistical significance (*p* < 0.05). Heat-tolerant group shown in green (*n* = 18) and heat-sensitive group shown in red (*n* = 25).

There was a low to moderate, positive correlation between measurement indicators (RR, BT, and MY) and THI with Pearson correlation coefficients ranging from r = 0.11 to 0.4 in the heat-tolerant group, and from r = 0.07 to 0.43 in the heat-sensitive group. There was a low positive correlation between VT and THI, with Pearson correlation coefficients r = 0.27 in the heat-tolerant group and r = 0.07 (no correlation) in the heat-sensitive group (Figure 4). Cows in the heat-tolerant group had a higher slope (to a unit increase in THI) in RR than cows in the heat-sensitive group; however, the slope of MY was the opposite, with a lower slope of MY in the heat-tolerant group. There were no significant differences in RR, BT, and VT (*p* > 0.05) between the heat-tolerant and heat-sensitive cows. However, the heat-sensitive group had significantly higher MY (*p* = 0.0007) than the heat-tolerant group (Figure 5).

### 3.3. Serum Biochemistry Indicators

There were significant differences between the heat-tolerant and heat-sensitive in the serum biochemistry of dairy cows (Table 5). The heat-tolerant group had significantly lower Glu (*p* = 0.02), COR (*p* = 0.02), 5-HT (*p* = 0.04), and IL-6 (*p* < 0.01) than the heat-sensitive group; whereas the heat-tolerant group tended to have lower HSP-90 (*p* = 0.10) and HSP-70 (*p* = 0.09) than the heat-sensitive group.

## 4. Discussion

A heat-tolerant animal may maintain homeothermy under high environmental heat loads, depending on the animal’s ability to balance thermogenesis and heat dissipation. Heat tolerance and sensitivity can reflect the animal’s ability to adapt to the thermal environment. Several measures have been proposed as the gold standard criteria for identifying the heat-tolerance and heat-sensitivity of animals, which include BT, VT, and RR [22]. Animal-based physiological indicators representing the animal’s response to environmental challenges are often used to assess the magnitude of HS in dairy cows [23]. Amamou et al. [14] reported the method of selecting cows with heat tolerance or sensitivity during HS. Herein, we identified cows that were tolerant or sensitive to HS based on the slope of the response in physiological and production traits against THI during the HS period. When defining heat-tolerance and heat-sensitivity, attention should be paid to both physiological and productive responses of cows to HS, as well as to the slope intensity relative to the mean population response. Al-Kanaan [24] expounded that the identification of heat-tolerant animals is useful only if these animals are able to maintain high productivity when exposed to HS conditions. In summary, our experiments assessed cows for heat tolerance or sensitivity by RR, VT, BT, and MY during periods of HS, and ultimately two groups of cows, with heat-tolerance or heat-sensitivity, were formed by clustering analysis. Moreover, we considered that cows qualified to be heat-tolerant by our work tended to have a higher slope of RR to THI and lower to almost no decay in MY compared to cows qualified to be heat-sensitive. It can be found that heat-tolerant cows are able to respond more quickly to the changes in THI to reduce the damage caused by heat stress.

Santana et al. [25] showed that when the level and slope of the cow’s response norms were positive and above, the animals became increasingly productive and positive plastic; the heat-tolerant group was consistent with these results. When animals are exposed to HS environments, heat dissipation is activated to adapt to high ambient temperatures. While other factors, such as skin and sweat glands, as well as coat type and condition, may affect thermoregulation in livestock, RR appears to have a greater contribution to heat abatement [26]. Animals enhance respiration to cope with high ambient temperatures to dissipate excess body heat through vaporization [27]. Cows promote blood circulation to dissipate heat through the skin by enhancing body muscle vitality to reduce peripheral vascular resistance and increase peripheral blood volume [28]. Skin is considered an important pathway for heat exchange, and BT is the result of regulating this exchange between the body’s core and skin through blood flow [29]. When they are unable to disperse excess heat by the respiratory tract and the skin surface, an animal starts using morphological, hematological, and biochemical mechanisms to regulate rectal temperature [30]. RR is a mode of thermo-regulation while VT is the result of thermal equilibrium [31]. In our study, heat-tolerant cows during HS were comparable to the aforementioned report. We consider that heat-tolerant cows were more heat-resistant than heat-sensitive cows. The heat-tolerant cows’ higher RR shows that they were able to cope with the thermal environment more quickly than the heat-sensitive cows [31]. The maintenance of MY levels in hot environments is associated with higher RR, and this was the major component of heat invulnerability in Holstein dairy cattle [14]. In our study, although there was no significant difference in RR between the heat-tolerant and heat-sensitive cows, the heat-tolerant group’s slope of RR to THI was much higher than that of the heat-sensitive group. The results obtained in our experiments show that heat-tolerant cows maintain MY by increasing RR more rapidly, and thus, obtain lower MY loss. However, their MY was significantly lower than that of the heat-sensitive group. This suggests that animals with lower MY will be the ones showing high heat tolerance, consistent with the results of the Caraban et al. [22]. In conclusion, we found that heat-tolerant cows adapt better to high heat environments because they rapidly stabilize their state by enhancing physiological mechanisms, especially, by increasing RR more rapidly to dissipate the excessive heat load. In contrast, heat-sensitive cows were delayed in responding to the thermal environment, and they were affected by HS more severely, with decreased MY.

As expected, correlations between the variables MY, RR, and BT, and environmental measures of THI were low to moderate and positive. These results are consistent with a previous study of acute HS in dairy calves where RR was found to be the most accurate indication of HS, in a shaded vs. non-shaded trial [32]. The R^2^ for the slope of RR and BT with THI in heat-tolerant and heat-sensitive dairy cows were higher than for other indicators, without taking into account other influencing factors. It emphasized the importance of RR in the process of identifying cows with heat tolerance and sensitivity. At the same time, we also believe that BT has high reliability in screening for heat-tolerant and heat-sensitive cows. This was further confirmed by our observations that the Pearson correlation between measurement indicators RR and BT and THI was higher than all other measures indicators, without taking into account other influencing factors. Thus, we consider that RR and BT are the most useful physiological indicators for determining a dairy cow’s heat sensitivity and they can be used for future heat tolerance selection.

Heat stress is well-documented to significantly affect a variety of physiological and hormonal indicators in dairy cows. Attia [33] detected significant differences in blood biochemical levels in animals after heat stress, most likely due to a metabolic shift in stressed animals to cope with the induced stress. In extreme stress, one of the main adaptive responses displayed by the animal is neuro-endocrine regulation [34]. Through a variety of molecular mechanisms, HS can cause a variety of physiological and hormonal responses [35]. The HPA axis regulates the thermoregulatory system in animals by releasing a variety of neurotransmitters and hormones. The stimulation of the HPA axis, which results in increased release of glucocorticoids such as cortisol, occurs when animals are stressed. These then influence physiological processes by triggering changes in intracellular receptors [36]. The amount of glucocorticoid production and stimulation affects the HPA axis function and may have an impact on the animal’s health [37]. Cortisol, the primary glucocorticoid in most mammals, including cattle, is a key factor [38]. Cortisol levels in the blood are frequently used as a valid stress biomarker to detect how animals respond to various levels of stress [39]. Our heat-tolerant cows showed lower cortisol levels, indicating that they had been exposed to less HS. Hepatic gluconeogenesis, which aids in the generation of glucose from non-carbohydrate sources and maintains energy metabolism to support life-sustaining activities, would also benefit from a rise in cortisol levels [34]. The study shows that cooled cows have lower glucose levels when compared to heat-stressed cows [40]. In our study, heat-sensitive cows showed results similar to heat-stressed cows. Heat-sensitive cows exhibited higher blood cortisol levels, which encouraged hepatic gluconeogenesis and boosted non-carbohydrate glucose synthesis, resulting in higher serum glucose levels. IL-6 is a cytokine known to increase inflammation in the organism [41]. Du et al. [42] reported that in the HS healing group, the levels of blood IL-6 were reduced. In our experiment, the heat-tolerant cows had the same result. The central nervous system regulates the reactions of thermoregulatory effectors, such as cutaneous circulation, brown adipose tissue, and sweat glands, to external temperature challenges; 5-HT is thought to play a key function in this core thermoregulatory system [43]. In the rat hypothalamus, short-term HS accelerates 5-HT metabolism and increases the expression of 5-HT receptor genes [44]. The heat-tolerant cows showed lower levels of 5-HT in our trials, presumably due to their low-stress levels, thus, demonstrating that heat-tolerant cows have a better coping response to HS. In general, heat-tolerant cows mitigated the effects of HS through the HPA axis, which regulates a variety of physiological and hormonal responses. Heat shock proteins (HSPs) are a large family of proteins that are highly conserved in evolutionary lines [45], and can be seen in the thermal tolerance and adaptation of organism cells to various stresses. These proteins have chaperone activity that ensures the folding, unfolding, and refolding of stress-denatured proteins [46]. In addition, HSPs play a crucial role in the adaptation of mammals, including cattle, to stressful conditions [47]. These molecular chaperones encompass several families, classified according to molecular weight, and play important physiological roles in helping to cope with heat stress. HSP-70 is the most temperature-sensitive and is induced by stressors [48]. Liu et al. [49] found that the expression or serum levels of HSP-70 did not change in the summer. Nanas et al. [50] reported that cortisol and HSP-70 were significantly lower in thermotolerant cows than in thermosensitive cows. Our study had findings that were similar to these results. We observed a trend of variation in HSP-70 between cows that are heat-tolerant and those that are heat-sensitive, which may be responsible for the different thermotolerance between heat-tolerant and heat-sensitive cows. HSP-90, one of the HSP family genes, is highly abundant in the endoplasmic reticulum, cytoplasm and nucleus, and is responsible for protein translocation under normal conditions [51]. It assists protein folding processes to form functional proteins [52]. It plays an important role in protecting organisms from stress caused by a range of stressors, including heat or cold shock, hypertonic stress, food deprivation, reduced oxygen levels, and heavy metals [53]. Dangi et al. [54] report showed that HSP-90 levels are highest during the summer HS period. Our research found that the heat-sensitive cows exhibited higher stress levels and HS had more influence on their metabolism, as well as their health, and it was further confirmed that the two groups of heat-tolerant and heat-sensitive cows obtained from our experiments differ in their ability to adapt to HS environments.

## 5. Conclusions

In conclusion, two groups of cows with differing heat sensitivities were formed by clustering analysis, using data from our experiments during periods of HS. The impact of HS is obvious in all studied cows, with heat-tolerant cows less affected than heat-sensitive cows. Heat-tolerant cows upregulating RR rapidly, and the HPA axis regulates the thermoregulatory system in animals by releasing a variety of neurotransmitters and hormones to increase adaptability to thermal environments. In addition, we consider that the slopes of RR and BT to THI could be used as reliable indicators to identify heat tolerance or sensitivity. Overall, accurate feeding management and monitoring of heat-tolerant and heat-sensitive lactating dairy cows are important for improving cow welfare and dairy production.

## Figures and Tables

**Table 1:** Ingredients and nutrient composition of experimental diets (% DM basis).

Item	Value
Ingredients	Content, %
Alfalfa	10.39
Oat hay	2.42
Dandelion	0.48
Whole corn silage	48.33
Cottonseed	2.90
Beet pulp	2.42
Ground corn	7.49
Pressed corn	9.43
Soybean meal	8.70
Rapeseed meal	1.69
DDGS ^1^	0.72
Extruded soybean	1.33
Mineral and vitamin mix ^2^	3.70
Nutrient composition	
DM, % of wet TMR	62.40
CP	17.06
EE	3.32
NDF	35.75
ADF	18.20
NEL/(MJ/kg)	6.11

^1^ DDGS, Distillers Dried Grains with Solubles. ^2^ Contained the following per kg of diets: VA 170,000 IU, VD 8000 IU, VE 19,000 IU, Ca 160 g, P 50 g, Fe 800 mg, Cu 680 mg, Mn 3500 mg, Zn 7500 mg, Se 80 mg, I 400 mg, Co 38 mg.

**Table 2:** Environmental, production, and physiological measurement and blood sample sampling.

Items	*n*	Date	Time
Temperature-humidity index (THI, Environment)	/	During the trial period	All day
Milk yield	43	During the trial period	08:00–08:40, 15:00–15:40, and 20:00–20:40
THI for each cow ^1^	43	During the trial period	08:00–11:30, and 13:30–16:30
Respiration rate	43	During the trial period	08:00–11:30, and 13:30–16:30
Vaginal temperature	43	During the trial period	08:00–11:30, and 13:30–16:30
Body surface temperature	43	During the trial period	08:00–11:30, and 13:30–16:30
Blood samples ^2^	20	At the end of the trial period	14:00–14:30

^1^ The THI corresponding to the proximity of each cow at the time of production and physiological measurements. ^2^ Determination of heat shock protein 90 (HSP-90), heat shock protein 70 (HSP-70), glucose (Glu), cortisol (COR), 5-hydroxytryptamine (5-HT), and interleukin 6 (IL-6), 10 cows randomly selected from each group after cluster for a total of 20 cows.

**Table 3:** Clustering results are based on the individual slopes of respiration rate (RR), vaginal temperature (VT), body surface temperature (BT), and milk yield (MY), with respect to temperature-humidity index (THI).

Items	Cluster					
	*n*	1 (Heat-Tolerant)		*n*	2 (Heat-Sensitive)	
		Mean	Standard Deviation		Mean	Standard Deviation
RR (bpm/THI)	18	2.03	0.18	25	1.73	0.20
VT (°C/THI)	18	0.02	0.01	25	0.02	0.01
BT (°C/THI)	18	0.06	0.004	25	0.06	0.004
MY (kg/THI)	18	0.26	0.26	25	−0.07	0.25

The results are obtained by clustering the results of the model.

**Table 4:** The R^2^ and root mean square error (RMSE) for predicting respiration rate (bpm = breaths per minute), vaginal temperature (°C), body surface temperature (°C), and milk yield (kg) using THI in dairy cows from heat-tolerant and -sensitive groups (tolerant means heat-tolerant group; sensitive means heat-sensitive group).

Model	R^2^	RMSE
Respiration rate-tolerant	0.15	17.44
Respiration rate-sensitive	0.16	17.75
Vaginal temperature-tolerant	0.04	0.41
Vaginal temperature-sensitive	0.005	0.41
Body surface temperature-tolerant	0.19	0.39
Body surface temperature-sensitive	0.18	0.40
Milk yield-tolerant	0.02	5.92
Milk yield-sensitive	0.04	6.47

**Table 5:** Heat shock protein 90 (HSP-90), heat shock protein 70 (HSP-70), glucose (Glu), cortisol (COR), 5-hydroxytryptamine (5-HT), and interleukin-6 (IL-6) concentrations of cows from heat-tolerant and -sensitive groups where *n* = 10/group.

Items	Groups		*p*-Value *
	Heat-Tolerant	Heat-Sensitive	
HSP-90 (pg/mL)	273.98 ± 28.29	307.11 ± 52.74	0.10
HSP-70 (pg/mL)	37.70 ± 7.59	43.49 ± 6.63	0.09
COR (ng/m)	19.50 ± 5.50	26.81 ± 6.71	0.02
GLU (mmol/L)	2.69 ± 0.32	3.00 ± 0.21	0.02
5-HT (pg/mL)	228.46 ± 74.47	302.00 ± 69.71	0.04
IL-6 (ng/L)	298.66 ± 65.92	403.63 ± 58.88	<0.01

* Significance was declared at *p* ≤ 0.05; tendency was declared at 0.10 ≥ *p* > 0.05.

## Data Availability

All data used in the current study are available from the corresponding author on reasonable request.
